# Orally‐administrated mitochondria attenuate pulmonary hypertension with the aid of erythrocytes as carriers

**DOI:** 10.1002/ctm2.1033

**Published:** 2022-09-23

**Authors:** Rui Xiao, Jie Liu, Shengquan Luo, Zhe Yu, Jiwei Zhang, Yankai Lv, Jiansha Li, Matthieu Ruiz, Jocelyn Dupuis, Qinghua Hu, Liping Zhu

**Affiliations:** ^1^ Department of Pathophysiology School of Basic Medicine Tongji Medical College Huazhong University of Science and Technology Wuhan China; ^2^ Key Laboratory of Pulmonary Diseases of Ministry of Health Tongji Medical College Huazhong University of Science and Technology Wuhan China; ^3^ Department of Pathology Union Hospital Tongji Medical College Huazhong University of Science and Technology Wuhan China; ^4^ Department of Pathology, Tongji Hospital, Tongji Medical College Huazhong University of Science and Technology Wuhan China; ^5^ Department of Nutrition Université de Montréal Montreal Canada; ^6^ Montreal Heart Institute Montreal Canada; ^7^ Department of medicine Université de Montréal Montreal Canada

1

**Dear editor,**

We demonstrated in this study that functional CAR peptide‐labelled mitochondria were detectable in venous blood and in lungs of rats after enteric encapsulation and oral administration, and were able to attenuate pulmonary hypertension in two different experimental models.

Pulmonary hypertension is characterised by pulmonary vasoconstriction and remodelling, resulting in increased pulmonary vascular resistance eventually leading to right heart failure and death. It is well known that hypoxia induces pulmonary vasoconstriction,^1^, ^2^ but causes systemic vessel vasodilation.^3^, ^4^ One explication for these discrepancies in terms of hypoxia response may be the function and structure heterogeneity of mitochondria in smooth muscle cells from pulmonary vessels compared to systemic vessels.^5^, ^6^ Our recent studies have shown that femoral artery smooth muscle cell‐derived mitochondria via intravenous injection can be transplanted into pulmonary artery smooth muscle cells (PASMCs), a process attenuating pulmonary hypertension.^5^, ^6^

Mitochondria transplantation for conditions associated with mitochondrial dysfunction is emerging as a novel therapeutic strategy. Previous studies have conducted mitochondrial transplantation mainly by intravenous,^5^, ^6^ tissue injection^7^ and nebulization.^8^ If mitochondrial transplantation could be performed orally, it would greatly improve its safety and convenience.

To address this issue and considering the biological specificity of mitochondria, we linked the CAR peptide, a cyclic peptide with cell‐penetrating properties and lung targeting properties,^9^, ^10^ to mitochondria via the mitochondrial outer membrane localization peptide. Five mitochondrial outer membrane localization peptides were screened to link CAR peptide to the surface of the mitochondrial outer membrane, and then labelled with FITC (Figure 1A). We found higher labelling efficiency for peptides ‐1, ‐3, ‐5 rather than peptides ‐2, ‐4 (Figure 1B). Therefore, the peptides‐1, ‐3, ‐5 were used in subsequent experiments.

**FIGURE 1:**
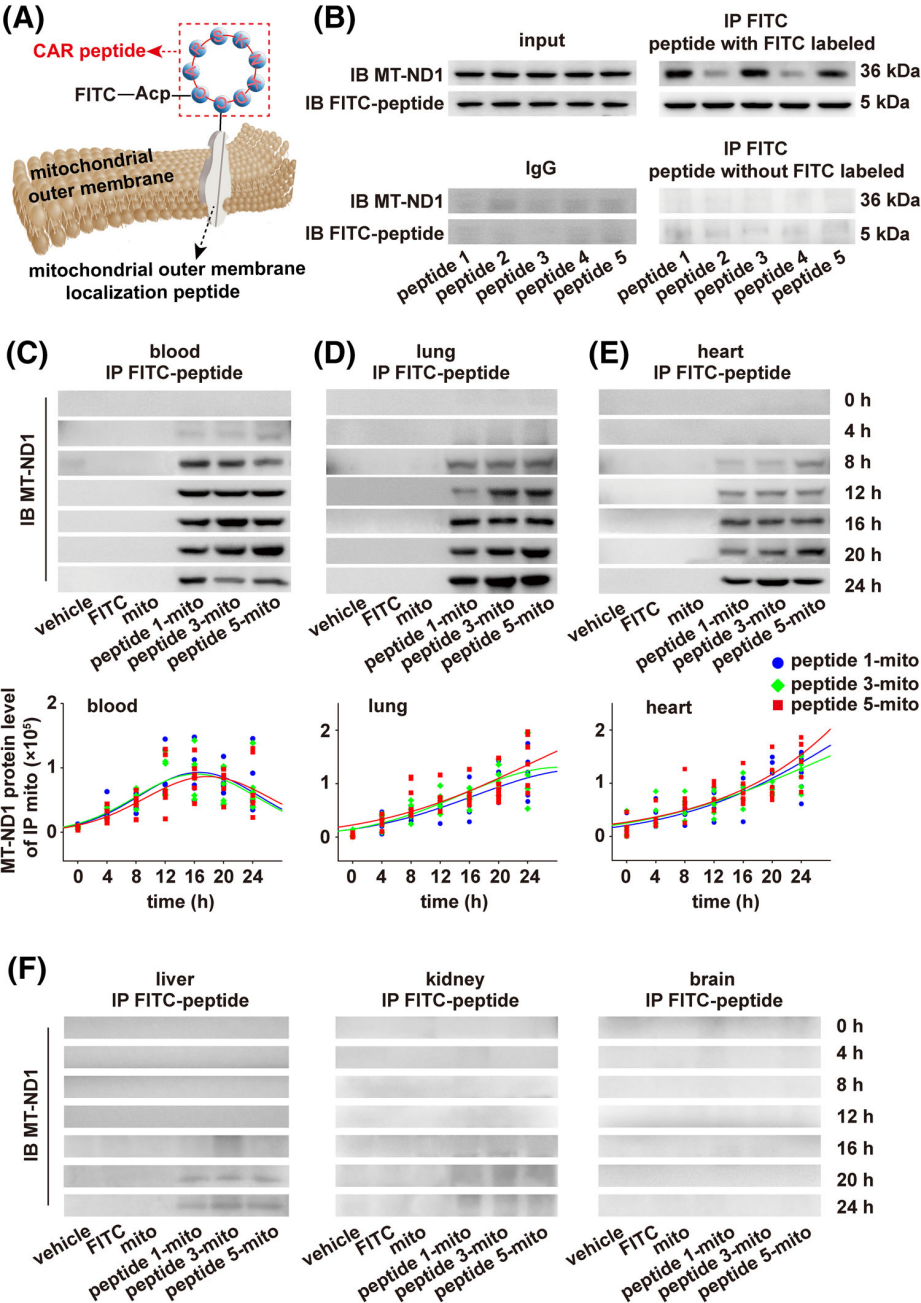
CAR‐labelled mitochondria were absorbed into venous blood and lung. (A) Schematic diagram of CAR peptide labelling mitochondria. Immunoprecipitation with FITC antibody in mitochondria lysates that co‐incubated with CAR peptides labelled with FITC (B, top right) or in mitochondria lysates that co‐incubated with CAR peptides without FITC label (B, bottom right), and immunoblotting with MT‐ND1 antibody and FITC antibody (similar results obtained from five independent experiments). (C–F) Immunoprecipitation with FITC antibody and IB with MT‐ND1 antibody at several time points from 4 to 24 h in blood, lung, heart, liver, kidney and brain. Regression curve of the amount of peptide 1 (blue)‐, peptide 3 (green)‐, peptide 5 (red)‐labelled mitochondria expressed in function of time in blood, lung and heart, *n* =5 for each following conditions: vehicle‐enteric capsule that encapsulated respiration buffer; FITC‐enteric capsule that encapsulated FITC; mito‐enteric capsule that encapsulated unlabelled mitochondria; peptide 1‐mito‐enteric capsule that encapsulated peptide 1‐labelled mitochondria; peptide 3‐mito‐enteric capsule that encapsulated peptide 3‐labelled mitochondria; peptide 5‐mito‐enteric capsule that encapsulated peptide 5‐labelled mitochondria

To assess whether CAR peptide‐labelled mitochondria could be absorbed into the blood through the small intestine, they were encapsulated to prevent any damage caused by acidic gastric secretions and administered to rats by gavage (see supplement). Labelled mitochondria were detectable after 4 h in the blood, 8 h in the lungs and heart (Figure 1C–E), ∼20–24 h with very small amounts in liver, while they were undetectable in brain or kidneys (Figure 1F).

We found that the number of labelled mitochondria in erythrocytes was much higher than that in plasma(∼4.64 fold) and white blood cells(∼8.91 fold, Figure 2A), and the number of mitochondria in venous erythrocytes increased significantly after oral administration of mitochondria labelled with peptides ‐1, ‐3, ‐5 compared with the vehicle group (Figure 2B).

**FIGURE 2:**
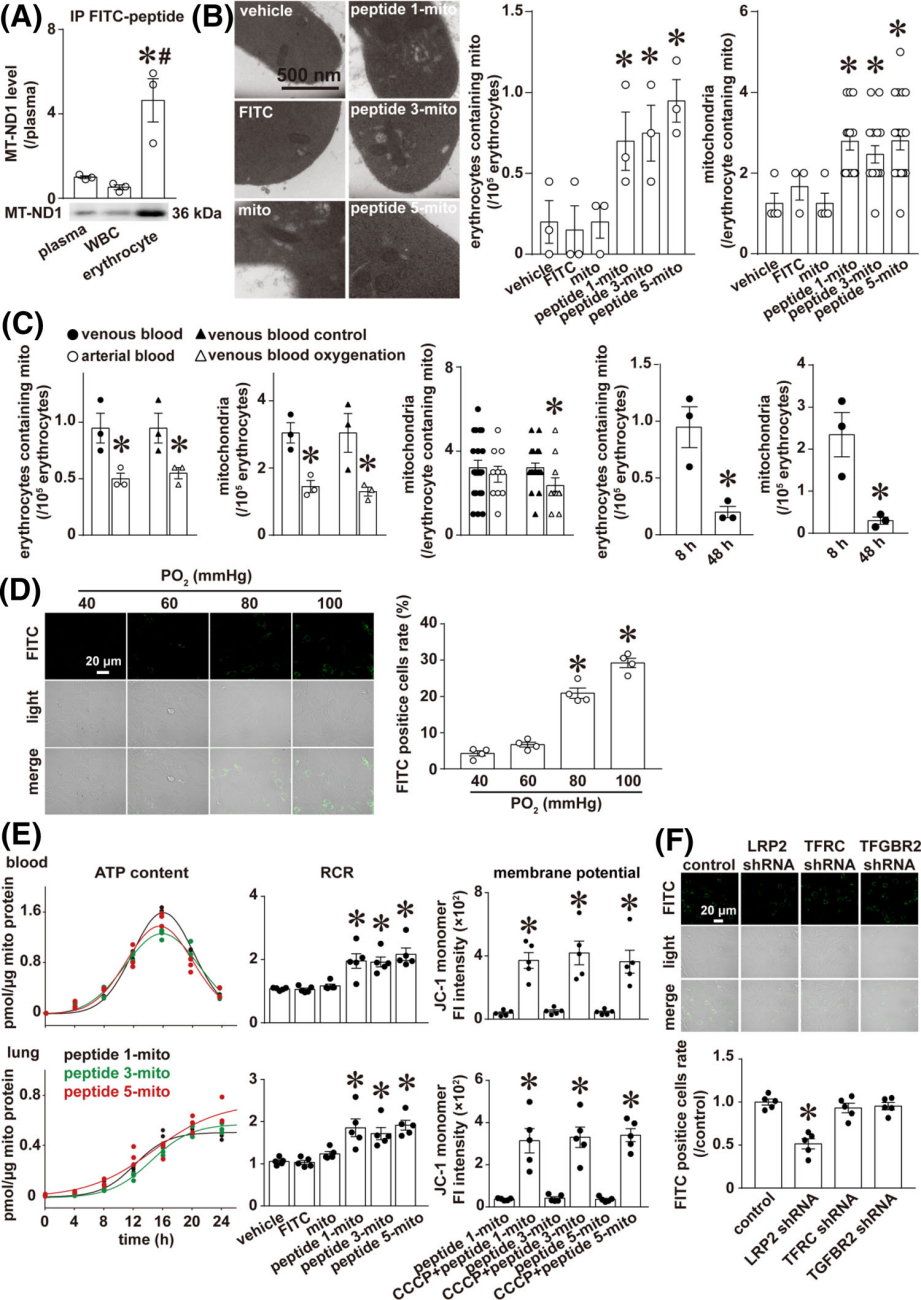
CAR‐labelled mitochondria absorbed into the blood via LRP2 were functional and mainly distributed in erythrocytes. (A) Representative image and corresponding histogram representation of IP with FITC antibody and IB with MT‐ND1 antibody of plasma, white blood cells (WBC) and erythrocytes; *n* = 3 rats, *p < .05 versus plasma, # *p* < .05 versus WBC, one‐way ANOVA followed by Student–Newman–Keuls analysis. (B) Representative images of mitochondria in the erythrocytes of each group, and corresponding histogram representation for (i) the number of venous erythrocytes containing mitochondria per 10^5^ erythrocytes and (ii) the average number of mitochondria in each venous erythrocyte among the erythrocytes containing mitochondria, *n* = 3 rats, *p < .05 versus vehicle, one‐way ANOVA followed by Student–Newman–Keuls post hoc analysis (the number of venous erythrocytes containing mitochondria per 10^5^ erythrocytes) or Kruskal–Wallis analysis followed by Dunn's post hoc analysis (the average number of mitochondria in each venous erythrocyte among the erythrocytes containing mitochondria). (C) Histogram representation of the number of erythrocytes containing mitochondria per 10^5^ venous or arterial erythrocytes; *n* = 3 rats, *p < .05 versus venous blood or venous blood control, and the Student's *t*‐test. The total number of mitochondria per 10^5^ venous or arterial erythrocytes; *n* = 3 rats, *p < .05 versus venous blood or venous blood control, and the Student's *t*‐test. The average number of mitochondria in each erythrocyte among the erythrocytes containing mitochondria in venous or arterial erythrocytes; *n* = 3 rats, *p < .05 versus venous blood or venous blood control, the Mann–Whitney rank sum test. Histogram representation of the number of erythrocytes containing mitochondria and the total number of mitochondria per 10^5^ venous erythrocytes at 8 and 48 h after gavage; *n* = 3 rats, *p < .05 versus 8 h, and the Student's t‐*t*est. The transwell experiment was undertaken as follows: The venous erythrocytes containing CAR‐labelled mitochondria on the top chamber and pulmonary artery endothelial cells (PAECs) on a circular glass coverslip at the bottom chamber were incubated for 24 h at different oxygen partial pressures. Then the circular coverslips were removed for microscopy examination of light field and FITC (CAR‐labelled mitochondria) fluorescence of PAECs. (D) Representative images and histogram representation of FITC‐positive PAECs on the circular coverslip. The ratio of FITC‐positive cells was calculated from the total number of cells and the number of FITC‐positive cells in three random areas of each coverslip from four separate experiments; * *p* < .05 versus 40 mmHg group, one‐way ANOVA followed by Student–Newman–Keuls post hoc analysis. The mitochondria extracted from the rat femoral artery were incubated with FITC‐labelled peptide for 1 h. The labelled mitochondria were coated with enteric capsules and orally administered to rats by gavage. Venous blood and lung were collected and mitochondria were extracted by immunoprecipitation with FITC antibody and functionally validated through evaluation of ATP content, mitochondrial respiratory control rate (RCR) and mitochondrial membrane potential at different time points in lysates of mitochondria or mitochondria suspension from venous blood and lung (E). *n* = 3 or 5 rats for each condition. The confluent intestinal villi endothelial cell (IVEC) monolayers on the top chamber were incubated with CAR‐labelled mitochondria, while pulmonary artery endothelial cells (PAECs) on a circular glass coverslip were placed at the bottom chamber. Twenty‐four hours later, the circular coverslips removed from bottom chamber of each transwell were examined for microscopy light field and FITC (CAR‐labelled mitochondria) fluorescence of PAECs. (F) Representative images and corresponding histogram representation of FITC‐positive PAECs on the circular glass coverslip placed in bottom chamber. *n* = 5/group, * *p* < .05 versus control shRNA, one‐way ANOVA followed by Student–Newman–Keuls post hoc analysis

Furthermore, the number of erythrocytes containing mitochondria was higher in venous blood than in arterial blood, and also higher in unoxygenated venous blood (venous blood control) than in oxygenated venous blood (venous blood oxygenation) (Figure 2C). The same trends were observed in the mitochondria number in erythrocytes (Figure 2C). The number of mitochondria per erythrocyte containing mitochondria was greater in unoxygenated venous erythrocytes than in oxygenated venous erythrocytes, while no significant difference was observed between venous erythrocytes and arterial erythrocytes (Figure 2C). The number of erythrocytes containing mitochondria in venous blood and the total number of mitochondria per 10^5^ venous erythrocytes were also superior at 8 h than at 48 h after gavage (Figure 2C). The transwell experiments on the erythrocytes charged with CAR‐labelled mitochondria highlighted an increased mitochondria release from erythrocytes with increased oxygen partial pressure (Figure 2D). These results suggest that mitochondrial release from erythrocytes may be regulated at least in part by oxygen partial pressure, and the release of mitochondria from erythrocytes may be in an All‐or‐None process in vivo, while in vitro the mechanism is somewhat between “All‐or‐None” with “partial” release.

CAR peptide‐labelled mitochondria were also obtained by immunoprecipitation with FITC antibody and were then functionally analysed for ATP production, respiratory control rate and membrane potential. The combined results showed that exogenous mitochondria absorbed in venous blood and lungs were functional (Figure 2E).

To determine the molecular mechanism of the absorption of mitochondria through the intestine, we screened intestinal protein candidates after interaction with exogenous mitochondria. A total of 2127 proteins were identified, and three of them were receptor proteins that have been known to mediate endocytosis or transcytosis, including TGF‐beta receptor type‐2 (TGFBR2), transferrin receptor protein 1 (TFRC) and low‐density lipoprotein receptor‐related protein 2 (LRP2). To explore which protein(s) mediates the transcytosis process of CAR‐labelled mitochondria, we implemented transwell experiments on the rat intestinal villus epithelial cells with CAR‐labelled mitochondria and found that LRP2, but not TFGBR2 or TFRC knockdown using shRNA, significantly reduced the transcytosis of CAR peptide‐labelled mitochondria (Figure 2F).

In rats under chronic hypoxia exposure or treated with a single intraperitoneal injection of monocrotaline (MCT), peptide‐5‐labelled mitochondria encapsulated with enteric capsules attenuated pulmonary hypertension as illustrated by the significant decrease of mPAP, PVR， RV/(LV+S) ratio and pulmonary artery wall thickness (Figure 3A–C). This improvement was associated with enhanced mitochondrial function in rat pulmonary arteries (Figure 3D,E). However, the benefits of CAR‐labelled mitochondria were lost with LRP2 silencing. Molecular mechanisms underlying the benefits of CAR‐labelling mitochondria also involved the reduction of extracellular calcium‐sensing receptors expression and related calcium signalling (Figure 4A–C), an important mediator of pulmonary hypertension as revealed in recent studies including ours.

**FIGURE 3:**
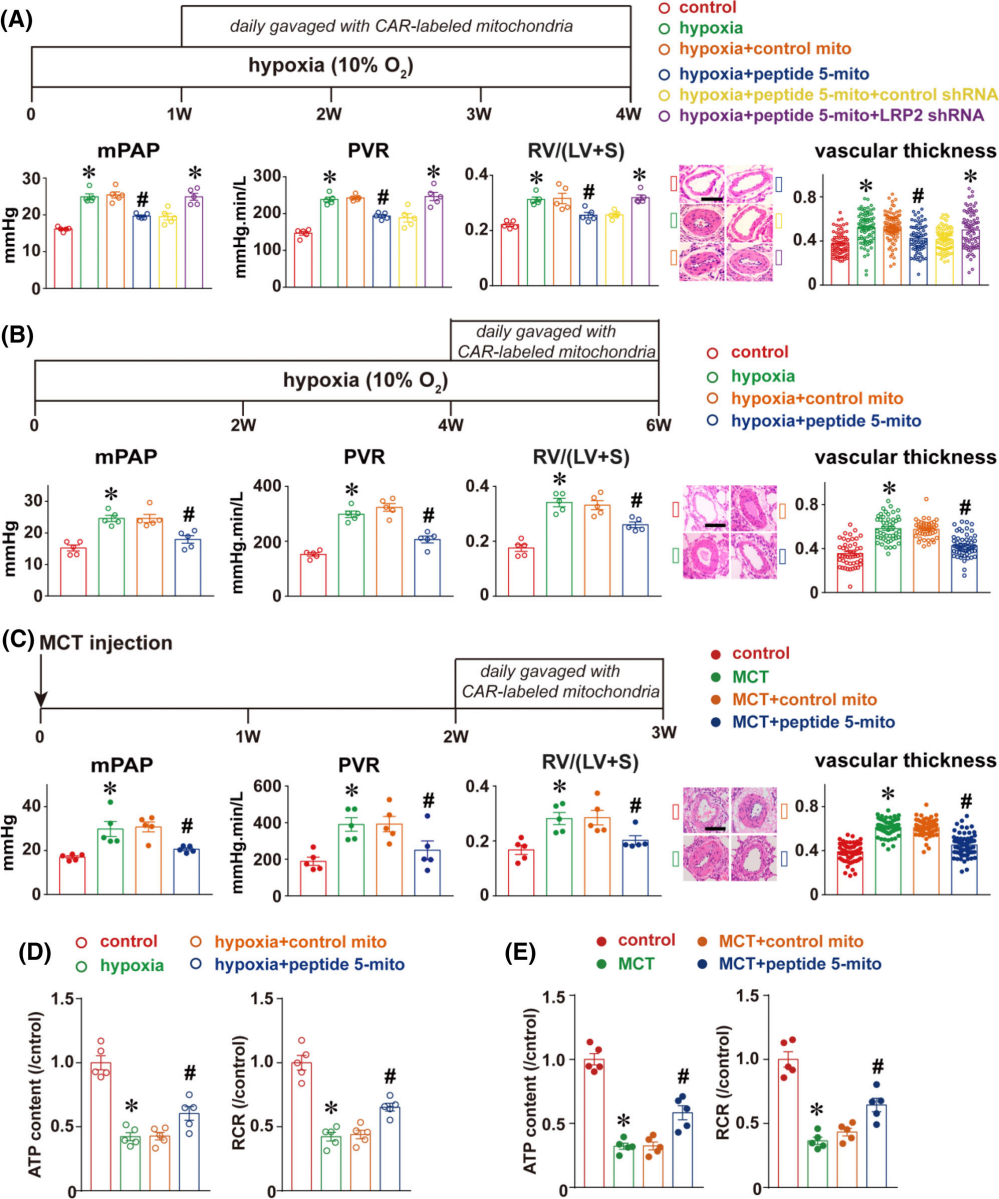
CAR‐labelled mitochondria attenuated hypoxia‐ and monocrotaline‐induced pulmonary hypertension. The procedure for evaluation of CAR‐labelled and orally‐administrated mitochondria on pulmonary hypertension in preventive mode in chronic hypoxia‐exposed rats (A) and therapeutic mode in both chronic hypoxia‐exposed (B) and monocrotaline (MCT)‐treated rats (C), and the summarised changes in key parameters, including mean pulmonary artery pressure (mPAP, left), pulmonary vascular resistance (PVR, middle), the Fulton index (RV/[LV+S], right), as well as representative HE stainings and histogram representation of pulmonary artery wall thickness (pulmonary arteries with diameter < 100 μm, *n* = 44–92 vessels). The mitochondria extracted from the rat pulmonary artery in each group were used to validate their functionality through evaluation of ATP content and mitochondrial respiratory control rate (RCR) in therapeutic modes in chronic hypoxia‐exposed (D) and MCT‐treated rats (E)

**FIGURE 4:**
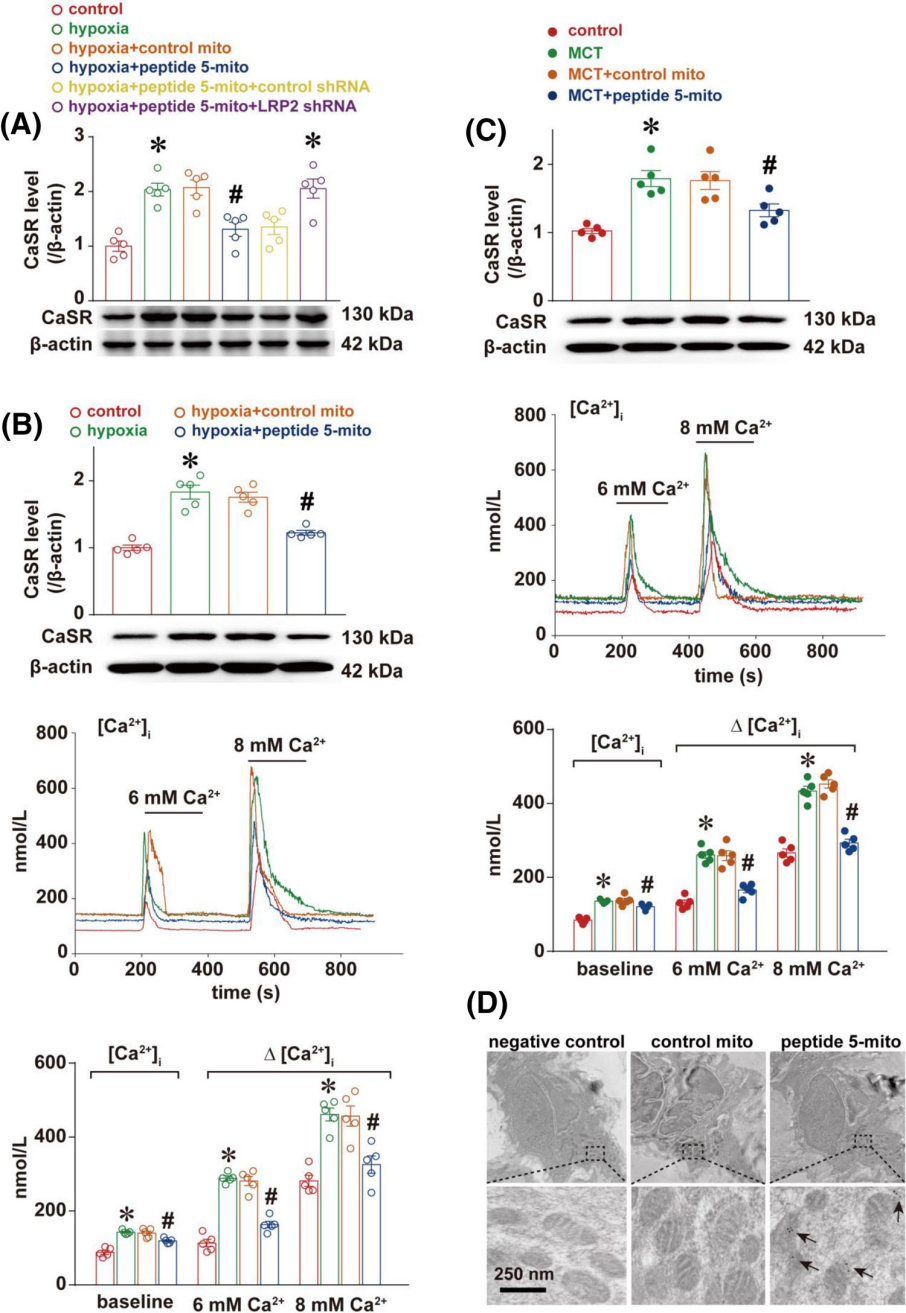
Molecular mechanism underlying the benefits of CAR‐labelled mitochondria is related to extracellular calcium‐sensing receptor. (A) The representative Western Blot and densitometric analysis of extracellular calcium‐sensing receptor (CaSR) in pulmonary arteries from chronic hypoxia‐exposed rats for evaluating the preventive effect of CAR‐labelled, orally‐administrated mitochondria. The representative Western Blot, densitometric analysis of CaSR in pulmonary arteries, representative responses and corresponding histogram representation of [Ca^2+^]_i_ in pulmonary artery smooth muscle cells (PASMCs) from chronic hypoxia‐exposed rats (B) and monocrotaline (MCT)‐treated rats (C) for evaluating the therapeutic effect of CAR‐labelled, orally‐administrated mitochondria. * *p* < .05 versus control, # *p* < .05 versus hypoxia or MCT, [Ca^2+^]_i_ of 13–17 PASMCs from separate five rats for each group, one‐way ANOVA followed by Student–Newman–Keuls multiple groups comparisons. (D) The representative immunogold electron microscopy showing the gold particle‐labelled mitochondria (peptide 5‐labelled mitochondria indicated by black arrow) distribution in rat pulmonary artery smooth muscle cells. Negative control was indicated for rats without any administration (left), control mito for rats administrated by gavage with mitochondria without peptide‐labelling (middle) and peptide 5‐mito for rats administered with peptide 5‐labelled mitochondria encapsulated with enteric capsules (right). In each condition, similar results were obtained from five separate rats

To confirm the localization of exogenous mitochondria in pulmonary artery, immunogold electron microscopy was conducted to examine pulmonary artery smooth muscle cells in rats, and gold particles were clearly identified in some PASMCs after intragastric administration of enteric‐coated capsules containing peptide 5‐labelled mitochondria (Figure 4D).

In conclusion, our data demonstrate that CAR peptide‐labelled mitochondria are absorbed from the intestine into blood after oral administration. The mechanism of mitochondrial uptake is associated with LRP2‐mediated transcytosis. We also showed that exogenous mitochondria absorbed into venous blood were mainly distributed into erythrocytes and were carried into the lungs. The release of mitochondria from erythrocyte was driven at least in part by increased oxygen partial pressure upon blood re‐oxygenation in lungs. Oral administration of encapsulated CAR peptide‐labelled mitochondria attenuated both hypoxia‐ and MCT‐induced pulmonary hypertension in rats. Our study provides a novel approach for easy and effective treatment of pulmonary hypertension, and reveals a new mechanism underlying exogenous mitochondria transportation and delivery through erythrocytes as carriers.

## CONFLICT OF INTEREST

The authors declare no conflict of interest.

## Supporting information

Supporting Information
