# Supplementary material for: Orally‐administrated mitochondria attenuate pulmonary hypertension with the aid of erythrocytes as carriers
Source: Clin Transl Med. 2022 Sep 23;12(9):e1033. doi: 10.1002/ctm2.1033 (PMC9505751; doi:10.1002/ctm2.1033)
Supplement: Supplementary file 1 — Supporting Information [file CTM2-12-e1033-s001.docx]

**Supplementary information**

**Materials and Methods**

**Ethical approval**

All procedures performed in animals were approved by the Institutional Animal Care and Use Committee of Tongji Medical College, Huazhong University of Science and Technology, and according to the guidance of the National Institutes of Health.

**Preparation of CAR-labeled mitochondria**

We screened 5 mitochondrial outer membrane localization peptides reported in the literatures [1-5]. The amino acid sequences are:

KTLLSLALVGACITLGAYLGHK [1];

TWQTVTIFVAGVLTASLTIWKKMG [~~1~~ 2];

KSGSGSKLLGLSLSLTLLGLLLLKAAK [3];

MKSFITRNKTAILATVAATGTAIGAYYYY [4];

NSMRYFKWGITRMILEATKPPIVLPIFAT [5];

The five peptides were conjugated with the CAR peptide and then labeled with FITC that generated the following five peptides:

Peptide 1: FITC-Acp-C*ARSKNKDC*-KTLLSLALVGACITLGAYLGHK

Peptide 2: FITC- Acp-C*ARSKNKDC*-TWQTVTIFVAGVLTASLTIWKKMG

Peptide 3: FITC- Acp-C*ARSKNKDC*-KSGSGSKLLGLSLSLTLLGLLLLKAAK

Peptide 4: MKSFITRNKTAILATVAATGTAIGAYYYY-C*ARSKNKDC*K-FITC

Peptide 5: NSMRYFKWGITRMILEATKPPIVLPIFAT-C*ARSKNKDC*K-FITC

Mitochondria were extracted from 0.2 g rat femoral artery according to the manufacturer procedure (mitochondrial isolation kit, ~~(~~C3606, Beyotime) and resuspended in 100 μL respiration buffer containing (mM): sucrose 250, KH_2_PO_4_ 2, MgCl_2_ 10, K^+^-HEPES buffer 20 (pH 7.2), K^+^-EGTA 0.5 (pH 8.0), glutamate 5, malate 5, succinate 8 and ADP 1. The 5 peptides were added at a concentration of 0.05 mmol/L and incubated for 1 hour at room temperature [6], before precipitation for 5 minutes after centrifugation at 13,000 g and 4 °C. The pellet was then resuspended in 100 μL respiration buffer. The extracted mitochondria were quantitated with Mito Tracker Green following our previous procedure [7, 8].

**IP and IB of CAR-labeled mitochondria**

CAR-labeled mitochondria (9×10^8^ for each rat) were encapsulated with an enteric capsule and daily administrated to rat by gavage. Six mL of venous blood ~~and~~ as well as lung, heart, liver, brain and kidney were collected from rat anaesthetized intraperitoneally with sodium pentobarbital at a dose of 30 mg/kg. The total mitochondria were extracted from the above tissues according to the manufacturer procedure (mitochondrial isolation kit, C3606, Beyotime). Mitochondria (~1.0×10^10^) isolated from tissue or venous blood were resuspended in 100 μL respiration buffer. Ten μL of agarose (sc-2003, Santa Cruz) was washed three times with PBS and mixed with the mitochondrial solution. The beads were then discarded by centrifugation after 1 hour incubation to remove components non-specifically bound to the beads. Five μg of goat polyclonal anti-FITC antibody (ab19224, Abcam) or goat IgG isotype (ab37373, Abcam) was added to the mitochondria solution and incubated overnight at 4 °C with gentle shaking. Then, agarose (sc-2003, Santa Cruz) was washed three times with PBS and added to the solution for incubation overnight at 4 °C with gentle shaking. After the immunoreaction, the solution was centrifuged at 1,600 g and 4 °C for 3 minutes and the beads were washed three times with PBS. Finally, 15 μL of SDS loading buffer was added to the agarose beads and boiled in boiling water for 5 minutes. After boiling and centrifugation at 1,600 g for 3 minutes, the supernatant was removed. Loading buffer was added to up to boiling for 10 minutes. The obtained sample was used for immunoblotting with rabbit monoclonal anti-MT-ND1 antibody (ab181848, Abcam) and goat polyclonal anti-FITC antibody (ab19224, Abcam) as described previously in detail [9].

**Transwell analysis**

Rat intestinal villus epithelial cells (IVECs) were cultured on the top chamber (Falcon Cell Culture Insert, CORNING) which contained polycarbonate membrane (pore size 0.4 μm) and transfected with shRNA against LRP2/TFRC/TGFBR2 using lipofectamine 2000 (Invitrogen). The cells were maintained in culture until reaching confluence. Rat pulmonary artery endothelial cells (PAECs) were ~~the~~ cultured in a circular slide (diameter 22 mm) at the bottom chamber. The FITC-CAR peptide-labeled mitochondria mixed with culture medium (2.35×10^8^ /mL) were added to the top chamber. The entire set of transwell were placed in an incubator and incubated at 37°C for 24 hours. The circular slide in bottom chamber was then removed for fluorescent microscopy examination of PAECs.

To evaluate the effects of oxygen partial pressure (PO_2_) on mitochondrial release from erythrocytes, CAR-labeled mitochondria (9×10^9^ for each rat) were encapsulated with an enteric capsule and administrated to rat by gavage as described above. After 16 hours, 6 mL venous blood was collected through a vacuum blood collection tube from rat anaesthetized intraperitoneally with sodium pentobarbital at a dose of 30 mg/kg. The venous blood was centrifuged at 1,600 g for 12 minutes and placed in an anaerobic incubator with PO_2_ at 40 mmHg (YQX-II Anaerobic Incubator, CIMO, Shanghai) to separate erythrocytes. The PO_2_ in the incubator was set at 40 mmHg by mixing air with N_2_ (3:1) yielding 5% O_2_ and continuously monitored using the isolated dissolved O_2_ meter and O_2_ electrode (ISO_2_, World Precision Instruments). Erythrocytes mixed with culture medium pre-equilibrated to 40 mmHg PO_2_ in the incubator. Then the mixture was added to the top chamber (Falcon Cell Culture Insert, CORNING) which contained polycarbonate membrane (pore size 0.4 μm). Rat PAECs were cultured in a circular slide (diameter 22 mm) at the bottom chamber with culture medium pre-equilibrated to 40 mmHg PO_2_. The entire set of transwell were placed in a cell incubator and incubated for 24 hours at four different oxygen partial pressures of 40, 60, 80 and 100 mmHg. The circular slide in bottom chamber was then removed for fluorescent microscopy examination of PAECs.

**ATP content detection**

Before and 4, 8, 12, 16, 20 and 24 hours after oral administration, total mitochondria were collected from venous blood, lung or pulmonary arteries following the above-mentioned procedure. Ten μL of agarose (sc-2003, Santa Cruz) was mixed with the mitochondria solution, the beads were then discarded by centrifugation after 1 hour incubation to remove components non-specifically bound to the beads. Then 1 μg goat polyclonal anti-FITC antibody (ab19224, Abcam) was added to the solution for incubation overnight at 4 °C with gentle shaking. Protein A/G PLUS-Agarose was washed three times with PBS and added to the solution for an additional incubation overnight at 4 °C with gentle shaking. After the immunoreaction, the solution was centrifuged at 1,600 g for 3 minutes at 4 °C to collect agarose beads. The agarose beads were washed with PBS for three times. The lysate from the ATP detection kit (S0026, Byotime) was added to agarose beads and mixed well. The supernatant was recovered for subsequent detection after centrifugation at 12,000 g and 4 °C for 5 minutes. One hundred μL of ATP test solution was added to the test well and left at room temperature for 5 minutes to remove any ATP background. Fifty μL of sample or standard was added to the well and mixed quickly. The relative light units (RLU) with a multifunctional microplate reader were measured (Synergy 2, BioTek). The standard curve was established with a series of 0.01, 0.10, 0.50, 1.00, 5.00, 10.0 μmol/L ATP for calibration of the ATP content of samples.

**Mitochondrial respiratory control rate detection**

CAR-labeled mitochondria collected from venous blood, lung and pulmonary arteries after oral administration as described above were suspended in PBS and subjected to respiratory control ratio (RCR) evaluation using the Clark-type oxygen electrode system (Hansatech Instrument Ltd., UK) and a Mitochondria RCR Assay Kit (Genmed. Arlington, MA, USA) following the manufacturer~~s’~~ procedures.

**Mitochondrial membrane potential (MMP) detection**

To evaluate MMP, CAR-labeled mitochondria were loaded with 10 µg/mL JC-1 (C2003S, Beyotime) at 37°C for 20 minutes. The green fluorescence intensity of JC-1 was detected by a fluorescence spectrophotometer (LS55, PerkinElmer) with the excitation and emission wavelength of 485 nm and 590 nm. Ten µmol/L CCCP was used here to depolarize MMP. JC-1 monomer yields green fluorescence at low MMP and the depolarization of MMP causes an increase of green fluorescence intensity of JC-1.

**Transfection of short hairpin RNA**

The shRNA against LRP2, TFRC, TGFBR2 and nonspecific control were purchased from Genechem (Shanghai Genechem Co., LTD., Shanghai, China). IVECs transfection was performed using lipofectamine 2000 (Invitrogen) according to our previous publication [10]. In brief, IVECs seeded on polycarbonate membrane were incubated at 37 ºC for 4 hours with 2 mL Opti-MEM (31985070, Gibco), which contained 2 µg shRNA and 5 µL lipofectamine 2000 reagent. Then, the medium was replaced with fresh DMEM and cultured for 48 hours before transwell assay. LRP2 knockdown in rats using lentivirus infection was conducted as previously described [11]. Briefly, the LRP2 shRNA was subcloned into lentiviral vector and packaged by Lenti-X HTX packaging system (Clontech). Lentivirus (1 mL, 10^8^ TU/mL) was injected into each rat through the tail vein.

**Extracorporeal oxygenation of venous blood**

A peristaltic pump (100 r/min, ZT600-1J, LongerPump) was connect to a membrane oxygenator (Xi'an Xijing Medical Products Co., Ltd., Xi'an, China) pre-filled with PBS and pumped with air or N_2_. Three mL venous blood in a 10 mL syringe was pushed into the inlet of the membrane oxygenator at a constant speed and passed through the membrane oxygenator within 2 minutes. The blood collected at the outlet of the membrane oxygenator was then centrifuged at 1,600 g for 12 minutes to recover the erythrocytes for a transmission electron microscope examination.

**Transmission electron microscopy**

A total of 2 mL of venous blood was taken from each rat, and 100 μL was taken for erythrocyte counting. In brief, the number of erythrocytes per mL of blood was obtained and then the blood volume of the rat was calculated based on the weight of the rat, and finally the total number of erythrocytes was calculated. The remaining blood was centrifuged at 1,600 g for 12 minutes and erythrocytes were collected, fixed with electron microscope fixation solution (G1102, Servicebio) for 4 hours at 4 °C, and rinsed with 0.1 mol/L PBS 3 times for 15 minutes. Then erythrocytes were fixed with 0.1 mol/L PBS containing 1% gallic acid at 20°C for 2 hours, and rinsed 3 times for 15 minutes each with 0.1 mol/L PBS. Then, the fixed erythrocytes were dehydrated for 15 minutes each time in 50%, 70%, 80%, 90%, 95%, 100%, 100% alcohol, 100% acetone and 100% acetone. The fixed erythrocytes was treated with i) acetone: 812 embedding agent (90529-77-4, SPI) (1:1, v/v) for 2-4 hours, ii) acetone: 812 embedding agent (1:2, v/v) overnight and iii), pure 812 embedding agent for 5-8 hours. Then fixed samples were inserted into a plate containing embedding agent and incubated at 37°C overnight. The sample was polymerized for 48 hours at 60 °C and then sliced on an ultra-thin microtome (Leica UC7, Leica) with a diameter of 60 nm. The sections were stained with 2% uranium acetate saturated alcohol solution for 15 minutes and then with lead citrate for 15 minutes, then dried at 20°C for 12 hours. Images were observed under the transmission electron microscope (HT7700, HITACHI).

**Estimation of mitochondria quantity in erythrocytes**

The evaluation of the mitochondria number in erythrocytes was assessed as follows: a slice of venous erythrocytes was made and loaded on a copper mesh with an area of 7.3 mm^2^. To estimate the total number of erythrocytes on the slice, we collected 3 fields of view each with an area of 2.55 × 10^-4^ mm^2^ and under the condition of 1,000 times magnification. We counted the number of erythrocytes, then calculated the erythrocyte density on the slice around 9.15 × 10^4^ mm^-2^, and the total number of erythrocytes on the slice around 7.3×9.15×10^4^=6.68×10^5^. Then we scan the whole slice under a magnification of 1,000 times to look for mitochondria. When oval or rod shape with high density was localized in erythrocytes ~~(as shown in figure~~ **~~2B~~**~~)~~ （因为组图时空间不够，该示意图在最终版letter figure 2中示意图已删掉）, the suspected mitochondria were confirmed under the condition of 10,000 times magnification. ~~This process is also shown in the top right diagram in figure~~ **~~2B~~**~~.~~ （因为组图时空间不够，该示意图在最终版letter figure 2中示意图已删掉） When the total number of mitochondria on each slice is determined to be x, the average number of mitochondria in 10^5^ erythrocytes is shown as x 6.68^-1^.

**Immunogold electron microscopy**

CAR-labeled mitochondria were encapsulated with enteric capsules and orally administered to rats. The lungs were fixed by in vivo infusion with electron microscopy fixative (1% paraformaldehyde + 0.1% glutaraldehyde), removed and placed in the electron microscope fixative to isolate pulmonary artery before being fixed for another 5 hours. The fixed pulmonary artery was dehydrated, embedded in epoxy resin, cut into 50 nm ultrathin sections, and placed on a nickel mesh. 1% H_2_O_2_ was dropped onto the wax plate. The slice was gently floating on the droplet at room temperature for 10 minutes, and then washed 3 times with PBS for 5 minutes each. Slice was incubated with 1% BSA for 30 minutes at room temperature, incubated with goat polyclonal anti-FITC antibody (1:100 dilution, ab19224, Abcam) at 4ºC for 24 hours, then at room temperature for 1 hour to allow the antibody to fully penetrate. Slice was washed 3 times with PBS each for 5 minutes, placed in PBS containing 1% BSA (pH 8.2) to provide an alkaline environment for the binding of the colloidal gold standard antibody, incubated with 5 nm gold-conjugated goat anti-rabbit IgG (1:100, diluted with PBS pH 8.2, 4120031001, Eton Bioscience) for 30 minutes at room temperature, and then washed 3 times with double distilled water each for 5 minutes. Finally, negative staining was performed using 1% uranyl acetate and 1% OsO~~4~~_4_ (in 0.1 mol/L PBS) and observed with transmission electron microscope (FEI Tecnai G2 20 TWIN). The negative controls either omitting the primary antibody or both the primary antibody and the secondary antibody were set to prove the specificity of staining.

**Intracellular Ca^2+^ Concentration ([Ca^2+^]_i_) Measurement**

The [Ca^2+^]~~i~~_i_ in pulmonary artery smooth muscle cells (PASMCs) was examined with the Ca^2+^-sensitive membrane-permeable fluorescent indicator fura 2-AM as we previously described in details [9, 11]. PASMCs isolated from rats were seeded on glass coverslips for about 12 hours, then the PASMCs adhered to the coverslips were washed three times with hepes-buffered saline (HBS) and loaded with 2 μM fura 2-AM (Invitrogen-Molecular Probes, Carlsbad, CA) at 37°C for 30 minutes. PASMCs were then washed three times with HBS and maintained for 30 minutes before experiment. To detect the CaSR activity, extracellular calcium concentration ([Ca^2+^]_o_) of the bath solution was increased stepwise from 2 to 6 and 8 mM during the assay.

**Animal model and treatment**

Pulmonary hypertension was induced in rats by chronic hypoxia and monocrotaline respectively as we previously reported [9, 12]. Briefly, SD rats (approximately 200 g) were exposed to 10% O_2_ for 4 weeks in a chamber or received a single injection of monocrotaline (60 mg/kg, i.p.). The oxygen content in the chamber was balanced with N_2_. CAR-labeled mitochondria or unlabeled mitochondria encapsulated with enteric capsule were administrated by gavage to each rat in 9×10^8^ daily dose in preventive and therapeutic modes as specifically described for each study.

Hemodynamic studies, evaluation of the Fulton index and pulmonary vascular remodeling were performed as we previous reported [8].

**Statistical Analysis**

The number of animals in each experiment was indicated in the figure legends. Data in all experiments were summarized as mean ± SE. When normality passed, Student’s t-test or One-way ANOVA analysis followed by Student-Newman-Keuls post-hoc analysis was used for two or more group comparisons respectively. When normality failed, Mann-Whitney rank sum and Kruskal-Wallis followed by Dunn’s post-hoc analysis were performed for two or more group comparisons respectively. A *p* < 0.05 was considered a significantly different.

**References**

1. Nguyen M, Branton PE, Walton PA, Oltvai ZN, Korsmeyer SJ, Shore GC. Role of membrane anchor domain of Bcl-2 in suppression of apoptosis caused by E1B-defective adenovirus. *J Biol Chem.* 1994; 269(24): 16521-16524.

2. Ausili A, de Godos A, Torrecillas A, Corbalán-García S, Gómez-Fernández JC. The interaction of the Bax C-terminal domain with membranes is influenced by the presence of negatively charged phospholipids. *Biochim Biophys Acta*. 2009; 1788(9): 1924-1932.

3. Wattenberg BW, Clark D, Brock S. An artificial mitochondrial tail signal/anchor sequence confirms a requirement for moderate hydrophobicity for targeting. *Biosci Rep*. 2007; 27(6): 385-401.

4. Li JM, Shore GC. Protein sorting between mitochondrial outer and inner membranes. Insertion of an outer membrane protein into the inner membrane. *Biochim Biophys Acta.* 1992; 1106(2): 233-241.

5. Herndon JD, Claypool SM, Koehle CM. The Taz1p transacylase is imported and sorted into the outer mitochondrial membrane via a membrane anchor domain. *Eukaryot Cell*. 2013; 12(12): 1600-1608.

6. Chang JC, Chang HS, Wu YC, et al. Mitochondrial transplantation regulates antitumour activity, chemoresistance and mitochondrial dynamics in breast cancer. *J Exp Clin Cancer Res.* 2019; 38(1): 30.

7. Zhou J, Zhang J, Lu Y, et al. Mitochondrial transplantation attenuates hypoxic pulmonary vasoconstriction. *Oncotarget.* 2016; 7(21): 31284-31298.

8. Zhu L, Zhang J, Zhou J, et al. Mitochondrial transplantation attenuates hypoxic pulmonary hypertension. *Oncotarget.* 2016;7 (31): 48925-48940.

9. Zeng X, Zhu L, Xiao R, et al. Hypoxia-induced mitogenic factor acts as a nonclassical ligand of calcium-sensing receptor, therapeutically exploitable for intermittent hypoxia-induced pulmonary hypertension. *Hypertension.* 2017; 69(5): 844-854.

10. Chen T, Zhu L, Wang T, Ye H, Huang K, Hu Q. Mitochondria depletion abolishes agonist-induced Ca^2+^ plateau in airway smooth muscle cells: potential role of H_2_O_2_. *Am J Physiol Lung Cell Mol Physiol.* 2010; 298(2): L178-L188.

11. Zhang J, Zhou J, Cai L, et al. Extracellular calcium-sensing receptor is critical in hypoxic pulmonary vasoconstriction. *Antioxid Redox Signal.* 2012; 17(3): 471-484.

12. Xiao R, Su Y, Feng T, et al. Monocrotaline induces endothelial injury and pulmonary hypertension by targeting the extracellular calcium-sensing receptor. *J Am Heart Assoc*. 2017; 6(4):e004865.
